# P-766. Patient Experiences of Deferring Treatment for Further Evaluation and Monitoring after a Trace Xpert Ultra Result

**DOI:** 10.1093/ofid/ofae631.961

**Published:** 2025-01-29

**Authors:** Caitlin Visek, James Mukiibi, Mariam Nantale, Annet Nalutaaya, Patrick Biché, Joowhan Sung, Francis Kayondo, Joab Akampurira, Michael Mukiibi, Rogers Kiyonga, Achilles Katamba, Emily A Kendall

**Affiliations:** Johns Hopkins University School of Medicine, Baltimore, Maryland; Walimu, Kampala, Kampala, Uganda; Walimu, Kampala, Kampala, Uganda; Walimu, Kampala, Kampala, Uganda; Johns Hopkins Bloomberg School of Public Health, Baltimore, Maryland; Johns Hopkins University School of Medicine, Baltimore, Maryland; Walimu, Kampala, Kampala, Uganda; Walimu, Kampala, Kampala, Uganda; Walimu, Kampala, Kampala, Uganda; Walimu, Kampala, Kampala, Uganda; Makerere University College of Health Sciences, Kampala, Kampala, Uganda; Johns Hopkins University School of Medicine, Baltimore, Maryland

## Abstract

**Background:**

For people with “trace” sputum results (PWTS) on Xpert MTB/RIF Ultra, further evaluation and monitoring rather than reflex TB treatment may be appropriate. Patients’ preferences regarding treatment deferral have not been explored.

**Figure:**
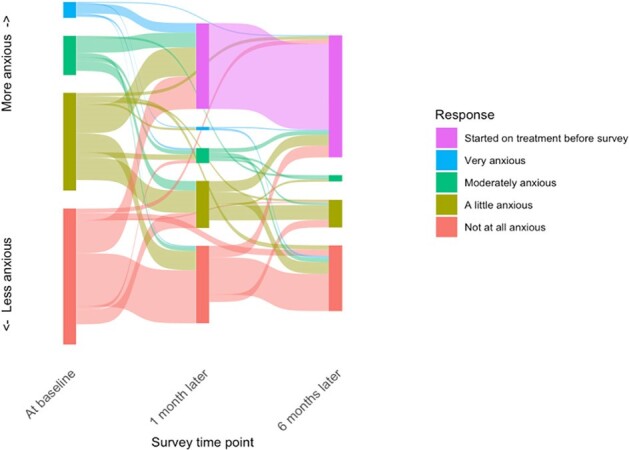

Reported anxiety among participants with trace positive sputum over 6 months of follow up

**Methods:**

We enrolled PWTS identified through community screening or clinic-based diagnostic evaluation, in Kampala, Uganda. They underwent extensive clinical, microbiological, and radiographic TB evaluation, and those with uncertain TB status were offered treatment deferral with periodic reassessment for up to 2 years. At 0, 1, and 6 months, PWTS were asked about their experience, including their perceived likelihood of having TB and associated anxiety, whether they would choose treatment if not being closely monitored, and the acceptability of extensive diagnostic testing. PWTS and matched Xpert-positive and -negative individuals were asked to choose, in a vignette, between more sensitive and more specific TB tests.

**Figure:**
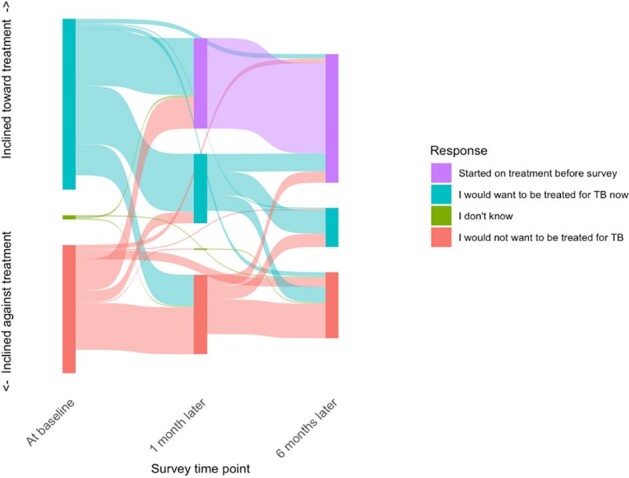

Reported inclination toward TB treatment among participants with trace positive sputum over 6 months of follow up

**Results:**

We enrolled 273 PWTS (128 from community; 145 from clinics), 217 people with positive results, and 252 with negative Xpert results. Among PWTS, treatment was recommended or started within 1 day of enrollment for 32/273 (12%), mostly based on recommendations of non-study clinicians in people with HIV, and within 1 month for 98/273 (36%), of whom 52/98 (53%) had Xpert or culture confirmation. Most PWTS (220/272, 81%) reported little to no anxiety at baseline and continued to report low levels of anxiety over time if they remained untreated, while many (25/52, 48%) PWTS with moderate or high initial levels of anxiety started treatment within 1 month (Fig 1). At baseline, 153/272 (56%) said they would seek TB treatment if monitoring were not offered, and most who remained untreated maintained the same preference over time (Fig 2). Most participants, including 65% of PWTS, preferred a highly sensitive test despite the risk of false positives when positive predictive value was 50%, and 47% favored sensitivity even when positive predictive value decreased to 17% (Table 1). PWTS rated laboratory and radiologic testing as valuable and minimally burdensome.

Table 1

**Figure:**
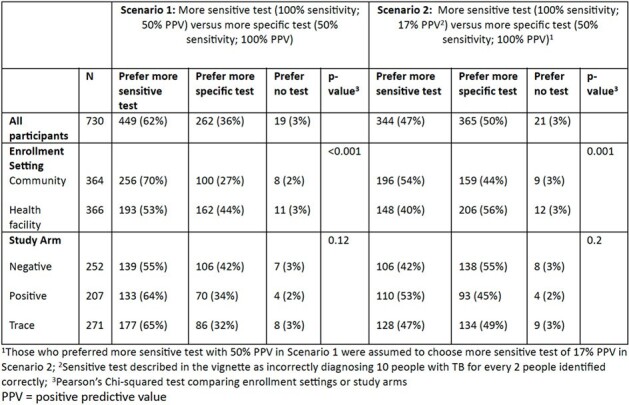

Participants’ preferences regarding TB test sensitivity versus specificity

**Conclusion:**

From the patient perspective, close monitoring rather than immediate treatment after a trace result may be acceptable.

**Disclosures:**

**All Authors**: No reported disclosures

